# PrP charge structure encodes interdomain interactions

**DOI:** 10.1038/srep13623

**Published:** 2015-09-01

**Authors:** Javier Martínez, Rosa Sánchez, Milagros Castellanos, Natallia Makarava, Adriano Aguzzi, Ilia V. Baskakov, María Gasset

**Affiliations:** 1 Instituto Química-Física “Rocasolano”, Consejo Superior de Investigaciones Científicas, Madrid 28006, Spain; 2 Centro Nacional de Biotecnología, Consejo Superior de Investigaciones Científicas, Madrid, Spain; IMDEA-Nanociencia, Madrid 28049, Spain; 3 Center for Biomedical Engineering and Technology, University of Maryland School of Medicine, Baltimore, MD 21201, USA; 4 Institute of Neuropathology, University Hospital of Zürich, Zürich 8091, Switzerland

## Abstract

Almost all proteins contain charged residues, and their chain distribution is tailored to fulfill essential ionic interactions for folding, binding and catalysis. Among proteins, the hinged two-domain chain of the cellular prion protein (PrP^C^) exhibits a peculiar charge structure with unclear consequences in its structural malleability. To decipher the charge design role, we generated charge-reverted mutants for each domain and analyzed their effect on conformational and metabolic features. We found that charges contain the information for interdomain interactions. Use of dynamic light scattering and thermal denaturation experiments delineates the compaction of the α-fold by an electrostatic compensation between the polybasic 23–30 region and the α3 electronegative surface. This interaction increases stability and disfavors fibrillation. Independently of this structural effect, the N-terminal electropositive clusters regulate the α-cleavage efficiency. In the fibrillar state, use of circular dichroism, atomic-force and fluorescence microscopies reveal that the N-terminal positive clusters and the α3 electronegative surface dictate the secondary structure, the assembly hierarchy and the growth length of the fibril state. These findings show that the PrP charge structure functions as a code set up to ensure function and reduce pathogenic routes.

The charge organization in proteins defines the inter- and intramolecular ionic interactions essential for folding, binding and catalysis^1^,^2^,^3^. Altered charges modify the structural dynamics, the aggregation, and amyloid formation propensity, among others, of elementary proteins processes in all protein conformational diseases^4^,^5^,^6^,^7^,^8^,^9^,^10^,^11^,^12^,^13^,^14^. In prion disorders, the prion protein (PrP), a two-domain chain with an N-terminal effector tail (FT) hinged to a C-terminal globular domain (GD), forms transmissible amyloids^15^,^16^. Although the information for both folding and misfolding is contained in the 90–231 sequence region, the regulatory role exerted by the charged FT and the pathogenicity of mutations related to exposed charges underline an intramolecular code that remains to be elucidated^10^,^17^,^18^,^19^,^20^,^21^,^22^,^23^,^24^,^25^,^26^,^27^,^28^,^29^,^30^.

PrP displays a peculiar charge pattern in its two-domain chain, with a polybasic FT and all acid residues located at the GD. The FT is composed of repeats flanked at either side by positively charged clusters, known as CC1 (residues 23–30) and CC2 (residues 101–110). Despite its intrinsically disordered tail, the protein undergoes ligand-induced folding and can wrap around the GD facing the α2–α3 exposed surface, which induces compaction of the α-fold^19^,^27^,^31^,^32^. Removing the CC1 cluster alters the α-fold stability, early nucleation steps, the polymer shape and the prion propagation efficiency^17^,^18^,^20^,^21^,^22^,^23^,^24^,^25^,^26^,^28^,^29^,^30^. CC2 plays a very active metabolic role, participating in both biogenesis and processing^33^,^34^. Importantly, its charge abrogation yields amyloids with PrP^Sc^-like features^35^. The spacing between CC1 and CC2 affects disease onset, GD stability and the FT effector function^28^,^30^,^36^,^37^. On the contrary, the GD contains all the acid residues of the chain. Among them, D144, D147, D178, E196 and E211 (numbered according to the human sequence) are involved in salt bridges that either stabilize (α1/α3) or link structural elements (β2 to α2 and α1 to α2/α3)^38^,^39^,^40^,^41^. Other residues, such as E146 and E152 in α1, D167 in β2-α2, and E200, D202, E207, E221 and E228 in α3, expose their side chains to solvent, thus defining electronegative surface clusters^39^. Of these charges, the structural D144 and D147 residues and their respective salt bridges stabilize PrP^C^, preventing conversion to protease resistance forms, whereas D178, E196 and E211 are prone sites for pathogenic mutations upon charge alteration^38^,^42^. Moreover, pathogenic mutations, such as E200K and Q217R, and the dominant negative E219K polymorphism alter the α3 surface electrostatic potential, inducing minor folding effects^39^.

To unveil the information encoded in the complementary solvent-exposed charge structure, we constructed several mutants consisting of charge reversions and inclusions and tested their effects on the properties of both the α-folded and fibrillar states. These modifications avoid the formation of non-polar surface patches resulting from abrogating charges and it solubility effect^43^. At the FT, both the CC1 and CC2 regions were modified by substituting their K residues with E (K2: K24EK27E, K4:K101EK104EK106EK110E and K6: K2–K4). At the GD, the α3 electronegative surface was perturbed by replacing E200 and E221 with K and by replacing Q217 and Q219 with charged R and K, respectively. We found that charges dictate a variety of structural and metabolic traits mostly through communication between domains. Effects such as the stabilization of the native α-fold, dictating the efficiency of the α-cleavage, attenuating the fibrillation propensity and yielding the most benign amyloids suggest that the charge design ensures PrP^C^ functions.

## Results

**Figure 1:**
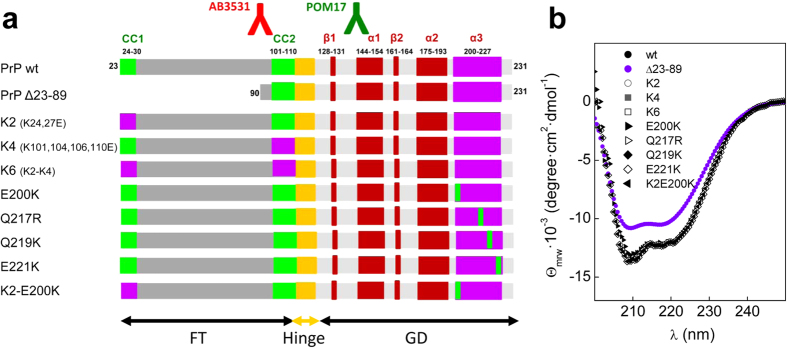
Charge structure of the PrP chain. (**a**) Modular organization of the PrP chain into an N-terminal domain (FT) hinged to a C-terminal globular domain (GD), displaying the location of the charged regions and their mutations that were considered in this study. Epitopes for Ab3531 and POM17 are depicted using the color codes of fluorescence microscopy. (**b**) Far-UV CD spectra in 10 mM MES pH 6.5 of PrP wt and of its charge and length mutants due to their α-folding.

To gain insight into the charge design of the PrP chain, we utilized reversion and insertion approaches using the rHaPrP (23–230) (PrP wt) as template (Fig. 1a). This choice preserved surface charge avoiding solubility effects resulting from charge abrogation^43^. At the FT, both the CC1 and CC2 charges were reversed (K2: K24EK27E, K4:K101EK104EK106EK110E and K6: K2-K4). At the GD, the α3 charge surface was modified by independent E200K, Q217R, Q219K, and E221K substitutions. Of these chains, K4 is equivalent to MoFBOM1^22^, E200K and Q217R are pathogenic mutations found in humans^44^,^45^, and Q219K represents the dominant-negative variant of MoPrP Q218K^46^. All of these chains were produced recombinantly, yielding cooperative folds with a predominantly α-helical secondary structure (Fig. 1b).

### PrP charge structure encodes an interdomain interaction promoting the α-fold compaction

**Figure 2:**
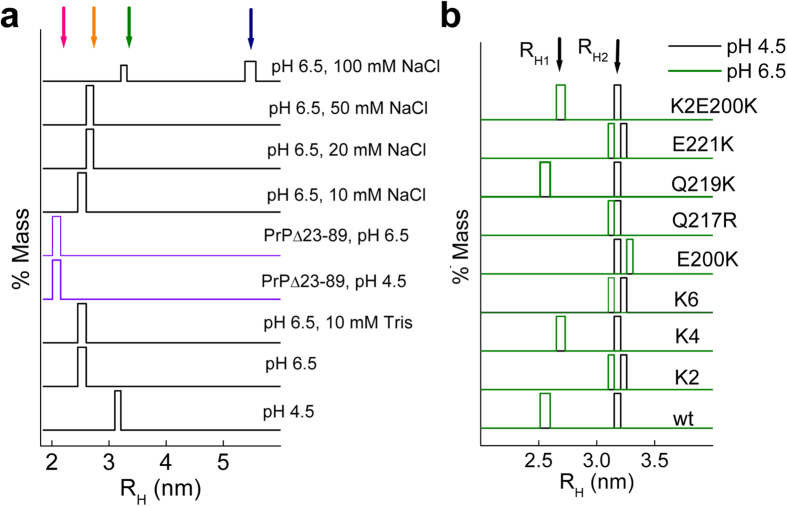
Effect of charges on the PrP hydrodynamic features. (**a**) Mass size distributions of PrP wt and PrPΔ23–89 as a function of pH and NaCl concentration. The theoretical values of R_H_ for spheres with similar MWs to PrP wt and PrPΔ23–89 are 2.25 and 1.96 nm, respectively. (**b**) Mass size distributions of PrP mutants in 10 mM NaAc pH 4.5 (black) and 10 mM Mes pH 6.5 (green). The measurements were performed at 25 °C using at least two different protein batches. Column widths show the standard deviation among measurements. Arrows at the top indicate the different R_H_ values.

Because charges are fundamental for intra- and intermolecular interactions, we probed the fold hydrodynamic properties using dynamic light scattering (DLS) (Fig. 2). To minimize insolubility interferences, measurements were performed using 15 μM protein concentrations at pH values of 4.5 and 6.5, for which open and closed PrP wt states have been reported, respectively^27^,^40^. Figure 2a shows that PrP wt yielded monodisperse species with R_H_ of 3.2 ± 0.1 (pH 4.5) and 2.6 ± 0.1 (pH 6.5) nm, whereas its GD as PrPΔ23–89 yielded an R_H_ of 2.1 ± 0.1 nm at both pH values^47^,^48^. Of these values, only the 2.6 nm for the PrP wt and 2.1 nm for the PrPΔ23–89 were comparable to the theoretical R_H_ values of globular protein spheres with the same molecular weight (2.25 and 1.96 nm, respectively), whereas the value of 3.2 nm for the PrP wt at pH 4.5 deviated from the ideal behavior, agreeing with the open state indicated via NMR. Importantly, the R_H_ values at pH 6.5 remained constant in the presence of 10 mM Tris used as a cation quencher, ruling out the effects of cation traces (Fig. 2a).

**Figure 3:**
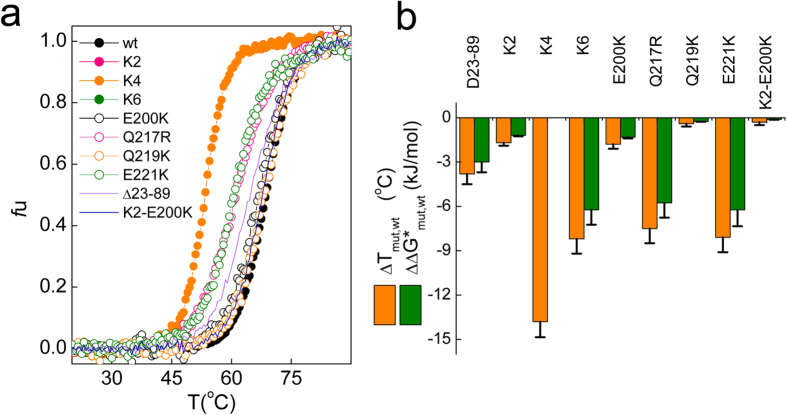
Effect of domain charges on the PrP stability. (**a**) Thermal denaturation of PrP wt and mutants. The unfolded fraction was calculated using the ϴ^222^ temperature function according to a two-state transition, as described^48^. **(b)** Differences in the unfolding temperature (ΔTm) and in the free energy of unfolding (ΔΔG*) induced by the charge mutations. ΔTm is the difference between the denaturation temperature of PrP mutant (PrP_mut_ or PrP_90−231_ wild type) and full length PrPwt (Tm_mut_ −Tm_wt_). Tm values were obtained as the midpoints of the temperature denaturation curves. ΔΔG* was obtained as ΔH_,wt _× (1−Tm_wt_/Tm_mut_), where ΔH_vH_ is the van´t Hoff enthalpy of PrP wt denaturation (255 kJ/mol). ΔΔG* < 0 indicates a destabilization of the mutant chain compared to the wt. Displayed data are the average of three independent measurements, performed with at least two different protein batches. Error bar represents the standard deviation (s.d.). Calculations for K4 were omitted given its irreversible denaturation.

At pH 4.5 all charge mutants yielded an R_H_ (approximately 3.2 ± 0.1 nm) similar to the PrP wt, ruling out perturbations in the open state (Fig. 2b). On the contrary, at pH 6.5, the R_H_ of K4 and Q219K decreased to 2.6 ± 0.1 nm, whereas the R_H_ values of K2 (both independently or combined with K4 as K6), E200K, Q217R and E221K remained unaltered. The lack of an R_H_ difference for these mutants suggests an impaired compaction and a structural role of CC1, E200, Q217 and E221 in such process. Given the hydrodynamic invariability of the GD and the pH-induced electropositive charge changes (His residues in octarepeats) in the FT, the impaired R_H_ reduction for the CC1, E200, Q217 and E221 mutants suggests that compaction involves an electrostatic interaction between the electropositive CC1 and the electronegative α3 surface, which is altered in Q217R. To further test this possibility, we constructed a K2-E200K mutant containing a double reversion and, after verifying its cooperative folding (Figs 1 and 3a), we tested its hydrodynamic properties. Figure 2b shows that the K2-E200K mutant behaved similar to the PrP wt, supporting the idea that the α-fold undergoes a compaction due to a long-range electrostatic interdomain interaction between the CC1 and α3 surface charges. The interaction driving the interdomain lock in monomeric PrP wt is weak, and NaCl concentrations above 50 mM provoke the emergence of species corresponding to the open (3.2 ± 0.1) and oligomeric (5.6 ± 0.2 nm) states (Fig. 2a), agreeing with the relevance of stabilizing factors such as coordinated cations^27^,^49^.

### Charge structure regulates α-fold stability through interdomain interactions in the native and denature states

To gain insights on the effect of the PrP charge structure on its thermodynamic stability, we analyzed the thermal denaturation curves, as shown in Fig. 3. It must be noted that measurements were performed in the absence of described interdomain cation stabilizers to avoid effects other than their complexation with His residues of the N-terminal octarepeats^27^,^49^. With the exception of K4, all of the thermal denaturations were reversible, as indicated by the recovery of at least 90% of the initial signal after cooling from the highest temperature. K4 denaturation was irreversible, and insoluble aggregates were detected upon cooling from the highest temperature (data not shown). Figure 3a,b show that the mutants with impaired compaction, such as K2, K6, E200K, Q217R, E221K and Δ23–89, unfolded with Tm values lower than that of wt PrP. This effect varied according to K6 ≈ E221K ≈ Q217R >Δ23–89> E200K ≈ K2, leading to decreases in the free energy of unfolding of about 1.2–6 kJ/mol. On the contrary, Q219K and K2-E200K, which undergo compaction, exhibited a PrP wt-like denaturation profile. Notably, the K4 mutant, exhibiting PrP wt-like hydrodynamic properties, yielded the most thermally labile fold, possibly due to its singular irreversible denaturation. Thus, with the exception of K4, these results indicate that altering the charges that mediate the interdomain lock decrease the native fold stability.

The destabilization pattern of the mutants precluding compaction agree with the effects described for the chains containing N-terminal truncations at pH > 6.0 and slightly differ from that described for PrP chains consisting in mutant GD moieties^20^,^21^,^37^,^50^,^51^,^52^ (Fig. 3b). By virtue of its additivity, the ΔG of folding of a two-domain protein (TD or full length) can be expressed as the sum of the contributions arising from the folding of each of domain (ΔG_FT_ and ΔG_GD_) and from their interaction (ΔG_FTGD_). Since the ΔG_FT_ and ΔG_FTGD_ are linked in PrP, their sum (ΔG*_FT_) can be calculated as ΔG*_FT _= ΔG _TD_ - ΔG_GD_. For the PrP wt and a GD consisting in PrPΔ23–89, ΔG*_FT_ corresponded to 3 ± 0.7 kJ/mol (−ΔΔG* in Fig. 3b), which agree with the 3.2 ± 1 kJ/mol value that can be calculated for GD consisting in PrPΔ32–89 chains^20^. For mutants exhibiting either identical GD as K2 or negligible effects on its free energy of unfolding as E200K^50^,^51^, which increasing GD breathing facilitates M213 sulfoxidation^53^, ΔG*_FT_ can be approximated to ΔG_TDwt_-ΔG_TDmutant_ difference (−ΔΔG* in Fig. 3b). This approach yielded values of 1.2 ± 0.2 and 1.5 ± 0.7 kJ/mol for K2 and E200K respectively. Taken together the values of PrPΔ32–89, K2 and E200K yielded an averaged estimation of ΔG*_FT_ of approximately 2 kJ/mol. On the hand, for Q217R the ΔΔG*_Q217R_ reported for the GD chains was −8.9 ± 2 kJ/mol^50^,^51^ and the calculated for the TD amounted to −5.8 ± 1 kJ/moly. As with Q217R, K6 reversing the charge of FT flanks provoked a similar ΔΔG* of −6.2 ± 1 kJ/mol. The higher destabilization of Q217R and K6 compare to PrP Δ23–89 suggested that in these mutants their charge changes affected interdomain interactions not only in the native state but also in their denature state^54^.

### Charges are gatekeepers of PrP fibrillation

**Figure 4:**
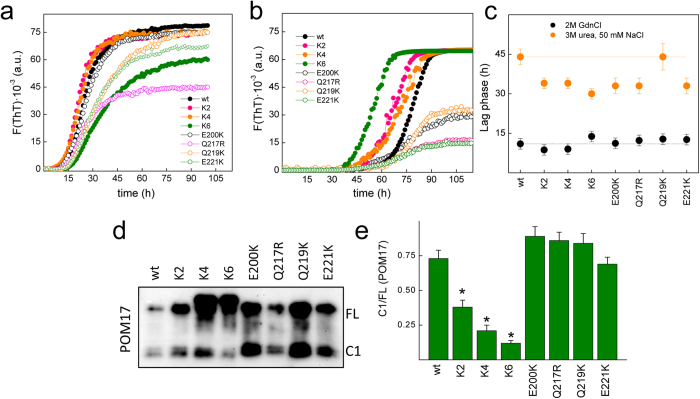
Charge changes modify the fibrillation propensity and processing of PrP. Time-dependence of ThT binding of the PrP wt and mutants (40 μM protein concentrations) in 50 mM MES pH 6.5 at 37 °C containing (**a**) 2 M GdnCl and (**b**) 3 M urea and 50 mM NaCl. The curves represent the average of three independent measurements, performed in triplicate. (**c**) Lag-phases of the fibrillation reactions of the PrP wt and mutants in 50 mM Mes pH 6.5 containing either 2 M GdnCl or 3 M urea with 50 mM NaCl. The depicted values correspond to three independent experiments, each performed in triplicate. (**d**) Western blot of PNGase-treated cell lysates of CHO cells transfected with PrP wt and the charged mutants. Detection was performed using POM17, and the positions of the full-length (FL) and N-terminal-truncated (C1 fragment) chains are depicted. (**e**) Variations in the C1/FL ratio of the PrP chains. Quantifications are the average of two independent transfection assays. Error bar represents the standard deviation (s.d.). *p < 0.01.

Although most PrP amyloids generated *in vitro* lack the infectivity and proteolytic signatures of PrP^Sc^, their formation models the chain propensity and the conformational changes required for GD self-assembly^55^,^56^. To test whether the charge structure, through either the α-fold stabilization described above or the initial self-assembly step, impacts the fibrillation, we performed time-dependent Thioflavin T (ThT) binding experiments using the various PrP chains at pH 6.5 and calculated the lag-phase as indicator of propensity (Fig. 4). To induce the required mild denaturation, we used either 2 M GdnCl (conventional ionic media) or 3 M urea containing 50 mM NaCl (low salt). As anticipated from the exposed character of the mutated charges and the high ionic strength of the media, fibrillation in 2 M GdnCl (Fig. 4a) yielded lag-phases that were roughly similar for all chains, with minor reductions (K2,K4) or enhancements (K6) (Fig. 4c). On the contrary, reactions in the low salt media containing 3 M urea and 50 mM NaCl revealed that with the exception of Q219K, all mutants form fibrils faster than the PrP wt as indicated by their higher lag-phases (Fig. 4b,c). Kinetic curves also showed that charge changes in the GD (E200K, Q217R, Q219K and E221K) decrease the ThT fluorescence increment linked to fibrillation predominantly under low salt (Fig. 4a,b), suggesting off-pathway events.

The observed reduction in the lag-phase in the low salt reaction media in K2, K4, K6, E200K, Q217R and E221K variants indicated that CC1, CC2 and the α3 electronegative caps function as fibrillation gatekeepers. Since K2, K6, E200K, Q217R and E221K impeded domain compaction and K4, with preserved compaction, lack the charges preventing the PrP fibril N-terminal packing^35^, together their effects suggest that charges regulate fibrillation propensity through their role in both the native fold compaction and the packing of critical regions for the seed stability. This charge effect agree with the increased propensity of PrP Δ23–89 compared to the full length chain^57^,^58^ and the dependence of anionic cofactors for functional fibrillation^59^.

### FT charges regulate the efficiency of C1 production

PrP processing into C1 chains is essential for the abrogation of prion formation and propagation, involving a complex cleavage within the interdomain hinge (around residue 110)^60^,^61^,^62^. To test whether the PrP charge structure plays a role in this processing, CHO cells were transfected with plasmids coding for PrP wt or its mutants, and the expressed chains were analyzed using POM 17 after PNGaseF digestion. Figure 4d,e shows that among the different PrP chains, only K2, K4 and their combination as K6 drastically reduced the efficiency of C1 production compared to wt PrP, which is in agreement with previous reports that showed impaired C1 production for mouse variants of K4 and PrPΔ23–31 in both Hpl cells and transgenic mice^22^,^25^. On the contrary, the mutants including modifications of the α3 surface charge impairing the interdomain lock, such as E200K, Q217R and E221K, yielded C1 levels similar to the PrP wt (Fig. 4d,e). Differences in the dependence of C1 production on the charge structure of either domain indicated that processing through the α-cleavage site is highly dependent on the FT charge structure but fairly independent of their role in driving the α-fold compaction. Thus, C1 production may require the recognition of the FT through its charge clusters by an unknown anionic ligand^52^,^63^,^64^,^65^, implying conditions with unlocked α-fold. These results suggest that the PrP charge structure regulates processing through its role in interactions.

### Charges impact the secondary to quaternary structure of the fibrils

**Figure 5:**
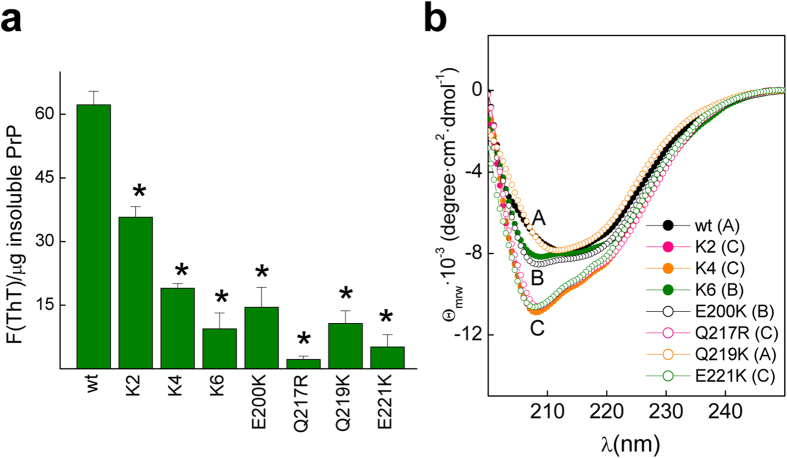
Properties of the PrP wt and mutant fibrils. (**a**) Specific ThT binding of the PrP wt and mutant fibrils. Typically 50 μM of fibrils in 10 mM ammonium acetate pH 5 were incubated for 10 min with ThT (15 μM) before fluorescence determination. ThT fluorescence intensities were corrected for the background (absence of fibrils) and divided by the protein amount in the pellet of a 12000 rpm 20 min centrifugation. The depicted data represent the average of two independent experiments performed in duplicate (*p < 0.01). (**b**) Far-UV CD spectra of the PrP wt and mutant assemblies in 10 mM ammonium acetate pH 5. The fibrils were formed in 50 mM MES pH 6.5 containing 2 M GdnCl at 37 °C under continuous rotation at 24 rpm, and then dialyzed against 10 mM ammonium acetate pH 5.

**Figure 6:**
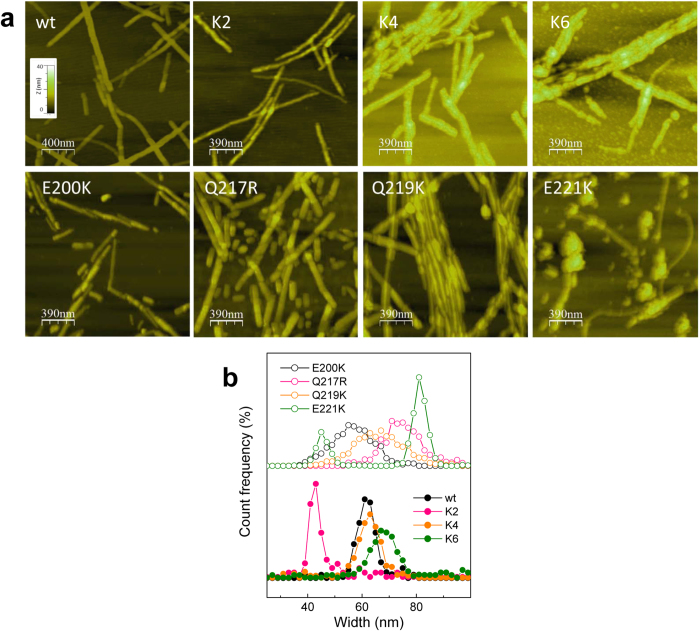
Topology images of the PrP wt and mutant fibrils. (**a**) AFM images of the PrP wt and mutant fibrils corresponding to the topology mode. The z-axis was fixed for all graphs, and the color scale is displayed as an insert in the PrP wt panel. (**b**) Histogram of the width distribution of the distinct PrP assemblies.

**Figure 7:**
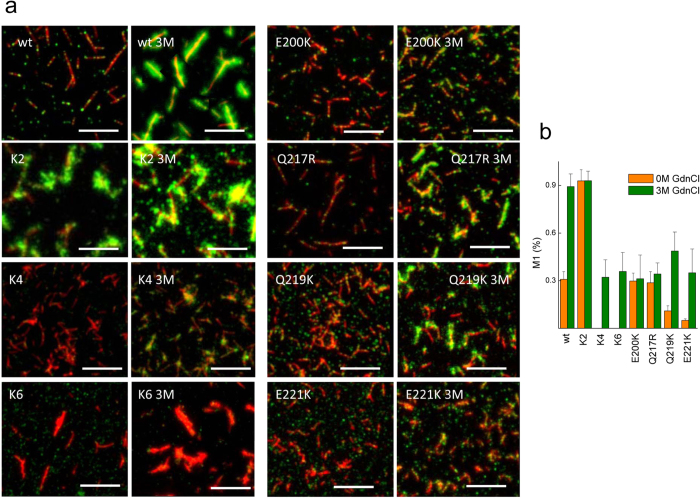
Surface reactivity of PrP wt and mutant fibrils. (**a**) Representative fluorescence images of the fibrils formed by the PrP wt and mutant fibrils stained with AB3531 (red) and POM17 (green). Pretreatment with 3 M GdnCl is indicated as 3 M. The white bar represents 5 μm. (**b**) Effect of charges on the variation of the POM17 overlap with AB3531 (Manders overlap coefficient) based on the denaturant concentration. Error bar represents the standard deviation (s.d.).

Similar to the α-folded wt PrP chain, variants with altered charge structure form fibrils that differ in their shape and proteinase-resistant core^21^,^35^. To gain insight into the effects of the charged design on the fibril structure and its properties, we used the reaction conditions yielding PrP wt S-fibrils with resolution for imaging analysis. S-fibrils, produced under slow orbital agitation as opposed to the fast rotation leading to the R-polymorph, are featured by a β-sheet-like far-UV CD spectrum along with a thin and curvy fibril topology that expose the 90–102 region while partially shield the POM17 epitope^66^ (Figs 5, 6, 7). It must be stressed that this fibrillation reaction was performed in the presence of 2 M GndCl, which abrogates the charge effects on the native α-fold and permits the assignation of the deviations from the wt behavior to differences in the assembly process. Fibrils formed by all PrP mutants displayed a reduced specific ThT binding, suggesting major structural differences (Fig. 5a).

Among the fibrils, only those formed by Q219K exhibited an S-like CD spectrum (Fig. 5b), whereas the assemblies formed by K2, K4, K6, E200K, Q217R and E221K displayed R-like CD spectra featuring a higher negative ellipticity, a minimum at 207 nm, and a shoulder at 217 nm of the β-sheet and turn structures (Fig. 5b). These data suggest a strict dependence of the S-fibril on CC1, CC2, and α3 surface charges. Because only the R-type fibril exhibited *in vivo* infectivity, the PrP charge structure appears to drive the fibrillation path to the less pathogenic S-type state^67^. The blockage of S-fibril type formation in K2, K4, K6, E200K, Q217R and E221K mutants also suggest that these charges may behave as sites for cofactor binding that, through their shielding, may shift the conversion route to the most infectious path^35^,^68^,^69^. Accordingly, in addition to known anionic cofactors which may function recognizing CC1 and CC2 regions may be molecules that exhibiting a basic charge would produce similar effects shielding the α3 surface charges , which would amplify the group of molecules functioning as cofactor in conversion^35^,^59^,^68^,^69^.

The Q219K substitution forms S-type-fibrils as PrP wt, but their assembly exhibited several differences. First, AFM imaging revealed that the Q219K fibrils, which were similar in dimension to those formed by the PrP wt, displayed a high degree of lateral association, yielding fibril layers rarely observed in PrP wt (Fig. 6). Second, the immunofluorescence analysis resolving fibrils in the μm range showed that in the wt and the mutant fibrils, the 90–102 (red, Ab3531 epitope) region is exposed along the fibril axis, whereas POM17 epitopes (140/145 region) were detected as discrete regularly spaced dots (green), which after treatment with 3 M GdnCl become disrupted in the wt fibrils but persist in the Q219K fibrils (Fig. 7a). These differences in the POM17/Ab3531 overlap both in the absence and presence of denaturant treatment are depicted in Fig. 7b.Thus, the lateral association and the attenuated surface reactivity of the Q219K fibrils may presumably contribute to the dominant-negative property of this charge insertion.

The K2 fibrils were thinner than the wt fibrils and tending to arrange into Y-shapes (Fig. 6). The immunofluorescence analysis revealed that the POM17 epitope was fully exposed in the absence of denaturant treatment (Fig. 7). On the contrary, K4 and K6 formed thick fibrils, indicating a higher assembly complexity. Of them, the peculiar stacking of the K6 assemblies resembled the aggregates of PrP27–30 rods^35^,^70^. Immunostaining revealed that the POM17 epitope was highly secluded in the K4 and K6 assemblies, resisting treatments, with distinct GdnCl concentrations (Fig. 7). These results indicate that CC1 and CC2 charges, in addition to allowing for S-type fibrillation, govern the evolution of the hierarchical assembly and the surface exposure of the region 140–145. CC1 appeared to work during the initial assembly step involving the shielding of the 140–145 region, whereas CC2 in a dominant fashion functioned during later assembly steps, compromising the discrete POM17 epitope exposure. These effects suggest that the FT charges modulate properties of the fibril state of the C-terminal domain.

Changes in the α3 charge structure resulted in R-type (Fig. 5b). AFM imaging revealed thicker fibrils compared to those of the PrP wt and images that were rich in short structures, rod-shaped in E200K and Q217R and spherical in E221K (Fig. 6). Interestingly, the E221K mutant revealed extremely thin, curvy fibrils emerging from the spherical aggregates (Fig. 6). The E200K and Q217R fibrils displayed a surface reactivity similar to that of the PrP wt but with green dots at reduced spacing, suggesting POM17 epitope location at the joint of the short rod-shaped structures (Fig. 7). In both assemblies, mild denaturing treatment increased POM17 staining but to a lesser extent that in the wt fibrils, suggesting differences in the folding of the 140–145 region. On the contrary, the E221K fibrils resembled those formed by K4, with a complete seclusion of the POM17 epitope and a discrete exposure upon treatment with 3 M GdnCl (Fig. 7). Taken together, these features suggest that the charges of the α3 primarily regulate the fibril length by either limiting growth or promoting fragmentation and the exposure of the POM17 epitope.

## Discussion

Here, we show that the PrP charge structure considered as solvent exposed contains information regarding interdomain interactions in both the PrP^C^-like conformation and the fibrillar state. In the PrP^C^-like formation, FT wraps the GD via the interaction between the N-terminal polybasic motive (CC1) and the α3 electronegative surface. This complementary charge interaction is relatively weak, and in the absence of stabilizers such bound Zn^2+^ the modifications provoked by the ionic strength or diminished surface electronegativity (single charge reversion or inclusions along the helix) impaired it. Notwithstanding, the interdomain interaction adds to the global fold a stabilization lower threshold of approximately 2 kJ/mol which interferes with fibrillation. Independent of this structural effect, the FT charge design also dictates the efficiency of cleavage between domains through the α-site indicating that the production of neuroprotecting fragments involves conditions with unlocked domains. In the fibrillar state, the PrP charge structure regulates the assembly process, dictating the secondary structure of the scaffold, the allowance of different (initial, intermediate and late) assembly steps, and the polymer length.

The sequence of proteins contains the protein’s functional information, including signaling mechanisms for maturation, sorting, covalent modification, and folding regulation, whereas other functions are encrypted and operate metabolically. The PrP sequence exhibits an unusual abundance of encrypted codes. The abundance of Met residues in the PrP chain allows for the translation of a minor chain that segregates out of the major secretory route, a structural regulation through a redox process, and a metabolically controlled substitution by SeMet^71^,^72^,^73^. Similarly, the His distribution entails a complex pH-regulated, cation binding trait in the FT that provides a diverse structural landscape with functional consequences^27^,^32^,^63^,^74^. Regarding charges, the complementary polybasic stretches in the FT and electronegative surfaces from the GD folding provide the basis for molecular compaction and its regulation by FT and GD ligands. Charge structures also participate in the fibrillation process, bestowing interdomain-dependent and domain-specific growth and assembly features.

The PrP charge structure seems to encode information for latent states with balanced ligand binding functions. The interdomain lock, functionally in non-transmembrane PrP formations, may sense changes in the length and flexibility of the FT, which depend on the number of repeats, the pH and cation binding^27^,^28^,^30^,^36^,^37^,^75^. Indeed, the interaction can be isolated either under partial protonation of the octarepeat His, as described here, or stabilized by Zn^2+^ binding^27^ to ensure ionic strength resistance. FT ligands that recognize its charge clusters regulate the cleavage of domains, suggesting that this process that monitors the dose of prion precursors uses as substrate the unlocked domain molecule. On the other hand, GD ligands precluding the lock by shielding the interacting motives (POM 4,10,19 and α-219–232) or by providing steric impediments (POM1) allow for FT toxic signaling^28^,^76^, suggesting that at the cell surface PrP^C^ may exit under the compact state. As in the GD the α3 surface constitutes the prion binding site, its shielding by the interdomain lock may also interfere with prion amplification by competing with seed binding^76^. Moreover, the interdomain lock prevents fibrillation of PrP by stabilizing the α-fold, which could be utilized in therapeutic intervention using the lock state as a target.

The charge structure code also operates in the fibrillation path, dictating properties that compromise propagation and toxicity. Despite the differences with PrP^Sc^, only R-type PrP fibrils exhibit *in vivo* propagation, which are only found in mutants with a modified charge structure^66^. The PrP charges appear to drive the chain into the fibril structures with attenuated pathogenicity and provide sites for cofactor action. Therefore, cofactors that bind to CC1 may regulate the initial steps, whereas cofactors that shield CC2, singly or combined with CC1, may drive the progression to the advanced assembly steps. These mechanistic hints agree with the mode of action of polyanions in previous conversion studies^35^,^59^. Moreover, the limits of assembly evolution due to FT charges are inversely correlated with the POM17 epitope exposure. Because this region contains the DWED sequence, forming part of the α1 in the α-fold and stabilized by salt bridges, it is tempting to postulate that the assembly involves its interaction with CC1. On the other hand, charged residues at either end of the α3, which are part of the β-core but not engaged in β-sheets, mainly govern the fibril length. Changing these charges reduces the length, either impeding growth or favoring fragmentation, which increases the seed concentration and consequently the propagation efficiency^77^. Thus, α3 mutations may strengthen their pathogenicity by optimizing their propagation through the seed concentration.

In conclusion, PrP charge design encodes several of structural and metabolic traits set up for ensuring function and diminishing pathogenic routes. These traits are: 1) shaping an interdomain lock, which prevents the FT from its toxic signaling and the GD from its prion receptor activity, 2) promoting the α-cleavage yielding anti-prion C1 chains and reducing the dose of chains with pro-prion activity, and 3) stabilizing the α-fold against conversion and if so allowing the less pathogenic assembly.

## Methods

### Production of PrP chains

rHaPrP (23–231) chains were produced from their pET11a constructs as described^48^,^78^, with minor modifications. Briefly, inclusion bodies were solubilized in 20 mM Tris pH 7.5 containing 6 M GdnCl, 05 M NaCl and 2.6 mM imidazole, and after column loading 6 M GdnCl was replaced by 8 M urea. rHaPrP(90–231) was produced from a pET15b construct using thrombin digestion to cleave the His-tag. The various mutants were produced via site-directed mutagenesis using the primers listed in Table S1. Before use proteins were equilibrated in the desired buffer (10 mM NaAc pH 4.5 or 10 mM Mes at pH 6.5, unless stated) by extensive dialysis and cleared by centrifugation before concentration determinations^48^.

### Dynamic Light Scattering

Dynamic light scattering (DLS) measurements were performed using a DynaPro spectroscatter (Wyatt Technology) with a 1.5-mm path length and a 12 μl quartz cuvette. The average of 20–25 acquisitions of buffers and protein solutions (15 μM protein concentrations) were filtered using a 0.1 μm Whatman Anodisc-3 filters. The hydrodynamic radii (R_H_) and mass proportions (%) of the species were derived from the autocorrelation data assuming a model of n-monodisperse globular proteins and using the software provided by the manufacturer^13^,^48^. Measurements were performed in triplicate using two different protein batches. The theoretical hydrodynamic radius (R_H_^T^) for PrP wt chain was calculated using 0.73 cm^3^ g^−1^ and 0.35 g H2O (g protein)^−1^ for the particle specific volume and the hydration, as described^47^.

### CD Spectroscopy

CD spectra were recorded in a Jasco-810 CD spectrometer with 15 μM protein solutions in either 10 mM Mes at pH 6.5 or 10 mM ammonium acetate pH 5.0 using a 0.1-cm cuvette^48^. Thermal denaturation experiments were performed following the ellipticity changes at 222 nm upon heating from 15 °C to 90 °C at a 1 degree/min heating rate and analyzed as a two-state transition as described^48^. Briefly, the changes in ϴ^222^ with temperatures were normalized to the fraction of unfolded protein (*f*_U_) using *f*_U_(T) = (ϴ^222^(T) − ϴ^222^_N_(T))/(ϴ^222^_U_(T)− ϴ^222^_N_(T)), where N and U refer to the native and unfolded states, respectively. The value of *f*_U_ was plotted as a function of temperature for the calculation of Tm and ΔHm. The experimental Tm values of mutants were converted into the apparent relative changes in free energy with respect to the wt protein (ΔΔG^o^_mut/wt_) using the equation ΔΔG_mut/wt _= ΔH_wt_ x (1 – Tm_wt_ / Tm_mut_), where Tm_wt_ and Tm_mut_ are the Tm values for the wt and mutant protein, respectively, and ΔH_wt_ is the denaturation van´t Hoff enthalpy of the wt protein.

### Fibril formation

The proteins in 5 mM MES pH 6.5 were cleared by centrifuging at 13200 rpm for 20 min at 4 °C and placed at 40 μM protein concentrations in 50 mM MES at pH 6.5 containing either 2 M GdnCl and 3 M urea with 50 mM NaCl. For kinetic analyses, 0.2-ml samples containing 15 μM ThT were placed in wells with a 3 mm glass ball. ThT binding was monitored at 37 °C, as described^13^,^73^. All measurements were performed in triplicate, and the experiments were repeated at least with two different protein batches. For imaging, fibrils were formed in 50 mM MES pH 6.0 containing 2 M GdnCl at 37 °C with continuous rotation at 24 rpm. After 100 h, the products were dialyzed against 10 mM ammonium acetate at pH 5.

### Atomic force microscopy

First, 2-μM fibril solutions were deposited onto freshly cleaved mica surfaces. After 10 min of adsorption, the samples were washed with H_2_O and dried with a stream of N_2_. AFM studies were performed using a MultiMode Veeco microscope with a 125-μm lateral range and a 5-μm vertical range equipped with a J-scanner and a NanoScope IIIa controller, using rectangular cantilevers with tetrahedral tips for the dynamic mode in air (Olympus, OMCL-AC240TS)^13^. The analysis was performed using WSxM (Nanotec).

### Fluorescence Microscopy

PrP fibrils were deposited onto Permanox 8-well Lab-Teks chamber slides and immunostained with rabbit anti-PrP Ab 3531 (1:1000, recognizes 90–102) and mouse POM17 anti-PrP Ab (1:1000, recognizes 140–145), followed by goat anti-rabbit and goat anti-mouse antibodies labeled with Alexa-488 and Alexa-546, respectively (Invitrogen/Molecular Probes, 1:1000). Fluorescence images were captured using an Axioplan Universal Microscope (Zeiss) equipped with a DFC 350 FX digital camera (Leica) and a 100x objective. Processing and analyses were performed using WCIF ImageJ software^79^. Manders overlap coefficients were determined as the amount of Ab3531 signal overlapped with the POM17 stain with at least 20 independent fibrils.

### Cell culture, transfections and processing analysis

Mutants were generated using pcDNA4.1-HaPrP(1–254) as a template (**Table 1S**)^80^. Transient transfections were performed using Chinese hamster ovary (CHO) cells and Fugene 6 as a transfection reagent (Roche), as described^80^. After 40 h, the cells were harvested via *in situ* lysis in a cold RIPA buffer (10 mM Tris-HCl pH 7.5, 100 mM NaCl 10 mM EDTA, 0.5% Triton X-100, 0.5% deoxycholate). The lysates were cleared via 5 min of centrifugation at 500 g, supplemented with 0.5 mM Pefabloc, and precipitated with 5 volumes of methanol at −20 °C. Samples were then centrifuged at 10000 × *g* for 30 min, and the pellets were re-dissolved in TNE buffer (50 mM Tris-HCl pH 7.5, 150 mM NaCl, 5 mM EDTA). Enzymatic digestions with PNGaseF (New England Biolabs) were performed for 1 h at 37 °C^80^. After digestion, the reactions were stopped by the addition of Laemmli buffer and PrP analyzed via immunoblotting using the POM17 antibody.

### Western blot analysis

The samples were resolved using SDS-PAGE (13.5% acrylamide gels) and electrophoretically transferred onto PVDF. The membranes were blocked for 1 h in 5% (w/v) non-fat dried skimmed milk powder in Tris-buffered saline containing 0.05% Tween 20. After incubation with mouse anti-PrP POM17 and HRP (horseradish peroxidase)-conjugated goat-anti mouse antibody (Sigma-Aldrich, 1:5000), the signals were developed using an ECL-Western-blotting reagent (Bio-Rad) and detected using ChemiDoc XRS equipment.

### Statistical analysis of experiments

Statistical analysis of experiments was performed either using paired Student’s t-test, for comparing two samples, or one-way ANOVA with Dunnett’s post-hoc test, for comparison of all columns to a control column. Results are displayed as the average of replicates ± s.d.

## Additional Information

**How to cite this article**: Martínez, J. *et al.* PrP charge structure encodes interdomain interactions. *Sci. Rep.* **5**, 13623; doi: 10.1038/srep13623 (2015).

## Supplementary Material

Supplementary Information
