# Supplementary material for: PrP charge structure encodes interdomain interactions
Source: Sci Rep. 2015 Sep 1;5:13623. doi: 10.1038/srep13623 (PMC4555102; doi:10.1038/srep13623)
Supplement: Supplementary Information [file srep13623-s1.pdf]

PrP charge structure encodes interdomain interactions

Javier Martínez[1], Rosa Sánchez[1], Milagros Castellanos[2], Natallia Makarava[3], Adriano Aguzzi[4], Ilia V. Baskakov[3] and María Gasset[1]*

[1]Instituto Química-Física "Rocasolano", Consejo Superior de Investigaciones Científicas, Madrid 28006, Spain.
[2]Centro Nacional de Biotecnología, Consejo Superior de Investigaciones Científicas, Madrid, Spain; IMDEA-Nanociencia, Madrid 28049, Spain
[3]Center for Biomedical Engineering and Technology, University of Maryland School of Medicine, Baltimore, MD 21201, USA.
[4]Institute of Neuropathology, University Hospital of Zürich, Zürich 8091, Switzerland.

Supplementary Information

Table S1. List of primers used in this study

Mutation	Template	Primer (forward)
K2	pETHaPrP(23-231)	5’-CATATGAAGGAGCGGCCAGAGCCTGGAG-3’
K2-E200K	pETHaPrP(23-231) K2	5’-GGAGAACTTCACGAAGACCGACATCAAG-3’
K2	pcDNA-HaPrP(1-254)	5´-GCCTCTGCAAGGAGCGGCCAGAGCCTGGAG-3
K4	pETHaPrP(23-231)	5´-GTGGAACGAGCCCAGTGAGCCAGAAACCAACATGGAGCACATGG-3´
K4	pcDNA-HaPrP(1-254)	5´-CAGTGGAACGAGCCTTCGGACCCAGAAACCAACATGGAGCACATGG-3´
K6	pETHaPrP(23-231) K4	5’-CATATGAAGGAGCGGCCAGAGCCTGGAG-3’
K6	pcDNA-HaPrP(1-254) K4	5´-GCCTCTGCAAGGAGCGGCCAGAGCCTGGAG-3
E200K	pETHaPrP(23-231)<br>pcDNA-HaPrP(1-254)	5’-GGAGAACTTCACGAAGACCGACATCAAG-3’
Q217R	pETHaPrP(23-231)<br>pcDNA-HaPrP(1-254)	5’-GTGTACCACCCGGTATCAGAAGGAG-3’
Q219K	pETHaPrP(23-231)<br>pcDNA-HaPrP(1-254)	5´-CAGATGTGTACCACCCAGTATAAGAAGGAG-3’
E221K	pETHaPrP(23-231)<br>pcDNA-HaPrP(1-254)	5’- CAGTATCAGAAGAAGTCCCAGGCCTACTAC-3’
